# Dietary diversity, undernutrition and anemia among rural adolescents in Sindh, Pakistan

**DOI:** 10.1186/s40795-026-01249-9

**Published:** 2026-01-17

**Authors:** Sana Sheikh, Atif Habib, Iqbal Azam, Rubina Barolia, Rahat Qureshi, Romaina Iqbal

**Affiliations:** 1 Department of Medicine, Aga Khan University, Karachi, Pakistan; 2 Department of Peadiatrics and Child Health, Aga Khan University, Karachi, Pakistan; 3 Department of Community Health Sciences, Aga Khan University, Karachi, Pakistan; 4 School of Nursing and Midwifery, Aga Khan University, Karachi, Pakistan; 5 Department of Obstetrics and Gynaecology, Aga Khan University, Karachi, Pakistan

**Keywords:** Stunting, Thinness, Anemia, Dietary intake, Adolescents, Rural, Low-middle-income country, Pakistan

## Abstract

**Background:**

Adolescence is the second window of opportunity to catch-up growth, and children can attain their full physical growth potential. However, optimum dietary intake is essential for this. In this study, we aimed to estimate dietary diversity, stunting, thinness, and anemia among unmarried adolescent boys and girls in selected communities of rural Sindh, Pakistan.

**Methods:**

A cross-sectional survey of 788 unmarried 10-19-year-old adolescents in rural Sindh was conducted. Adolescents were interviewed using a food frequency questionnaire. Daily intake of at least 5 out of 10 food groups was labelled as having minimum dietary diversity following the Minimum Dietary Diversity for Women (MDDS-W) guide. Less than 2 standard deviations for z-scores of height for age and BMI for age were labelled as stunting and thinness, respectively. Anemia was defined as Hb less than 12 gm/dl for girls and for boys between the ages of 10–15 years. Cut-off of < 13 gm/dl was used for anemia in boys more than 15 years of age. The Cox-proportional algorithm was used to analyze the associated factors of stunting, thinness, and anemia and prevalence ratios with 95% confidence interval were estimated.

**Results:**

Our study found that < 1% of adolescents achieved minimum dietary diversity (MDD). There was a higher daily intake of sugar-sweetened beverages (SSB) and bread, and daily intake of meat, eggs, and nuts was among < 1% of the participants. The sub-optimum dietary intake was reflected as high rates of stunting 31.9%, thinness 18.0%, and anemia 68.2% in our sample. Stunting and anemia were significantly higher among girls compared to boys (35.8% vs. 28.0%; 82.1% vs. 54.7% respectively). In sex-stratified analysis, the age of the adolescent and wealth quintiles were associated with stunting among girls. No other variables were associated with stunting, thinness, or anemia among girls and boys.

**Conclusion:**

The prevalence of low dietary diversity and anemia was alarmingly high in adolescents and calls for immediate attention. The high burden of stunting shows chronic undernutrition and missing the second window of opportunity during adolescence to gain potential adult height.

## Introduction

Consumption of a variety of food i.e. dietary diversity is a surrogate for sufficient nutrients intake and healthy growth in early years of life [1]. Adolescence (10–19 years of age) is an energy demanding phase for physical growth of body. A diet deficient in macro or micronutrients can affect the growth acceleration in this phase resulting in stunting, thinness, and deficiency of important minerals and vitamins required for growth [2]. A recent meta-analysis, reported 1.7 times odds of anemia with inadequate dietary diversity in adolescents [3]. Physiologically it is explained that acute shortage of macro and micro nutrients in diet can cause thinness and anemia and chronic insufficiency in quantity and quality of diet leads to stunting [3].

A diet higher in staples, refined carbohydrates, and low in fruits and vegetables among adolescents has been reported globally [4]. Most of the current literature is from high income countries and lack of literature on dietary intake among adolescents in low-middle-income countries (LMICs) has been highlighted consistently. However, the available literature from LMICs, found that foods rich in vitamins and minerals such as meat, poultry, eggs, dairy, and fruits and vegetables are consumed by a low proportion of adolescents [5]. A review of literature from Pakistan, reported high intake of sweets and confectionaries and low intake of vegetables and fruits in 5-18-years of school going individuals [6]. The factors influencing dietary quality encompass area of residence, race, ethnicity, education and socioeconomic status of the household, age, and gender of individuals, and their social status [7]. Higher level analysis showed that high-income countries have better diet quality hence better nutritional indicators than LMICs [8]. Males overall have better dietary intake than females [9]. Therefore, literature suggests a complex interplay of sociodemographic factors, dietary intake, and undernutrition [10, 11].

South Asia has the highest number of underweight adolescents (20% girls; 33% boys) [12]. However, the data on adolescent nutritional status is mainly reported for females. A systematic review in South Asia found 45 studies on females and only six on boys [13]. The National Nutrition Survey (NNS) 2018, Pakistan, was the first large-scale assessment of the nutritional status of male adolescents in the country. The survey reported 34.7% stunting and 21.3% thinness in rural male adolescents [14]. Pakistan is ranked among the countries with the highest burden of maternal and child undernutrition [15]. Despite high undernutrition burden, there is comparatively limited literature available on dietary intake in adolescents. A global review of characteristics of dietary intake data found that, Pakistan predominantly has data on school going adolescents from urban areas [16]. The country has about 20 million 10-19-years of age individuals who are not attending school and lack of data on this group will mask the true burden of undernutrition. Considering high rates of undernutrition in adolescents, it is imperative to examine the dietary intake and other associated factors of undernutrition and anemia comprehensively in both boys and girls of 10–19 years of age. Hence, we conducted this study to estimate the dietary diversity and prevalence and factors of undernutrition i.e. stunting, thinness and anemia among rural adolescents in selected areas of Sindh, Pakistan.

## Methods

### Theoretical framework

We pinned our study on food framework given by Pieters et al. on determinants of food and nutrition insecurity and adapted it using the literature on adolescent undernutrition [17]. The adapted framework demonstrates that multiple sociodemographic factors can influence dietary intake and consequently, undernutrition among individuals. In the context of adolescents, characteristics like gender, age, earning capacity, and their education can determine their chance to have an advantage of better nutritional status than others. Additionally, the education and employment status of parents, number of family members, and socioeconomic status of the household are also important factors for dietary quality and nutritional status [7, 10, 11].

### Study design and data collection

We conducted an analytical cross-sectional study in three union councils of sub-district rural Hyderabad in Sindh from February 2016 to December 2018. Sindh province was selected due to the highest number of underweight boys compared to other provinces [18]. The Pakistan Social and Living Standard Measurement Survey, on dietary diversity in different provinces of Pakistan found that household dietary diversity has improved in all the provinces from 2014 to 2019, except Sindh where a decline in dietary diversity was observed [19]. Moreover, another comparison of provinces on multidimensional poverty (access to education and healthcare, basic living standards, and monetary status) found Sindh to have second highest poverty after Balochistan [20]. Considering the above factors, the study was conducted in Sindh, urban and rural areas. The findings from urban areas have been published previously [21].

We recruited 799 unmarried (1:1 boys and girls) 10–19 years of age participants living in the study area for at least past 6 months. The boys and girls with self-reported co-morbidities which could have affected their nutrition status such as chronic renal/cardiac disease, cancers of all types, thalassemia major or other blood disorders, and diabetes mellitus were excluded. The written consent was obtained from all the participants as explained below. The study was reviewed by the Ethical Review Committee of Aga Khan University and approval was obtained under ERC# 3960-Obs-ERC-15.

Eligible participants were recruited through a door-to-door community-based survey through non-probabilistic sampling. Before interviews, two written consents were taken at each household. Mothers provided written consent for household level interview and for adolescents’ interview written consent (≥ 18 years of age) or assent was (< 18 years of age) taken from adolescents. For younger than 18-year participants, written consents of mothers were also obtained. Mothers of participants were interviewed for sociodemographic information about the household such as the education and occupation of the parents and the number of people in the household and for household-level food insecurity (FI).

Trained research staff interviewed girls and boys for their education, and adolescent’s FI. Detailed methods and results of FI assessment for household and adolescents have been published earlier [22]. The dietary intake data on a validated food frequency questionnaire with 91 food items was administered to adolescents [23]. Lastly, their height, weight, and hemoglobin were measured. Tanita digital weight scale and Shorr board were used to measure weight and height respectively. Participants were asked to remove shoes for both height and weight measurements. Two readings for weight and height were taken. If there was a difference of more than 0.1 kg in weight or > 2 centimeters in height, then a third reading was taken and an average of the three readings was recorded. Hemoglobin (Hb) was measured through HemoCue machine [24]. Finger prick was done and standard field procedures for Hb testing through HemoCue were followed [25]. All sharp items were disposed off in the danger bins safely.

### Statistical analysis

Sample size was calculated using WHO sample size calculator keeping prevalence of stunting in rural area 47% [26], anticipated difference in the prevalence of stunting 5%, sample size came out to be 783.

### Variables description

Undernutrition categorization: Hb less than 12 gm/dl for girls 10–19 years and boys of 10–15 years were categorized as anaemic. Cut-off of < 13 gm/dl was used for anemia in boys more than 15 years of age [27]. To estimate stunting and thinness anthropometric measurements were entered in WHO AnthroPlus 1.0 and gender and age-specific z scores were calculated. Z score of less than 2 standard deviations for height for age was labeled as stunted and for BMI for age as thinness [28].

Socioeconomic status: Wealth quintiles (poorest, poor, middle, rich, and richest from 1st to 5th quintile) were developed using an asset-based approach using the information of household possessions such as TV, motorcycle, refrigerator, housing characteristics (type of construction), and source of water and sanitation facilities. The details of this method have been published earlier [21].

Minimum dietary diversity: It was assessed by using Minimum Dietary Diversity-Women (MDD-W) guide which is recommended by FAO and has been validated in Pakistan [29]. As we did not find any relevant tool for adolescents’ dietary diversity, we used MDD-W as a reference. Multiple studies including a comparative report of 18 countries on adolescent nutrition also used MDD-W to measure adolescents’ dietary diversity [30, 31].

Following the Minimum Dietary Diversity-Women (MDD-W) guide, ten food groups were made and participants who consumed at least 5 out of 10 food groups were categorized as having minimally adequate dietary diversity [32, 33] i.e. MDD was labelled ‘Yes’ if the daily diet included ≥ 5 food groups, MDD was labelled ‘No’ when daily diet has < 5 food groups. The food groups included [1] Grain, roots, and tubers (GRT) [2] pulses [3] Nuts and seeds [4] Dairy [5] Meat, poultry, and fish [6] eggs [7] dark leafy greens and vegetables [8] vitamin A-rich fruits and vegetables [9] Other vegetables [10] and Other fruits [32].

Other co-variates: Based on descriptive statistics, mother and father education were categorized as illiterate (no education) and literate (at least 1 year of schooling). To determine the effect of age of the adolescent on their nutritional status, age was divided into early (10–14 years) and late adolescence (15–19 years) [34].

### Descriptive and inferential analysis

Descriptive results i.e. frequency and percentage are reported for dietary intake for girls and boys. Due to the sparsity of data in the group consuming ≥ 5 food groups, the variable was not included in the inferential analysis. The gender difference between consumption of different food groups and MDD was assessed through chi-square test. The p-value of < 0.05 was considered significant. Simple and multiple Cox proportional regression algorithm was used for inferential analysis at univariate and multivariable levels. Crude and adjusted prevalence ratios with a 95% confidence interval (CI) were reported for univariate and multivariable analyses respectively. A p-value of less than 0.20 at univariate level was considered eligible for multivariable analysis. Lee and Chia suggested prevalence ratio (PR) estimation as the preferred method for the strength of association instead of odds ratio (OR) in cross-sectional survey design [35]. OR can be reported for rare outcomes but for outcomes with more than 10% of the prevalence, OR provides inflated estimates and smaller p-values thus incorrectly concluding the association between two variables [36, 37]. Separate models were developed for factors associated with adolescent stunting, thinness, and anemia. Models were developed after checking the assumptions of Cox algorithm. Multicollinearity between independent variables was determined before adding them in the multivariable model. Possible interactions between variables for synergistic or antagonistic effects were also checked. Data analysis was done with SPSS version 25.0.

## Results

**Fig. 1:**
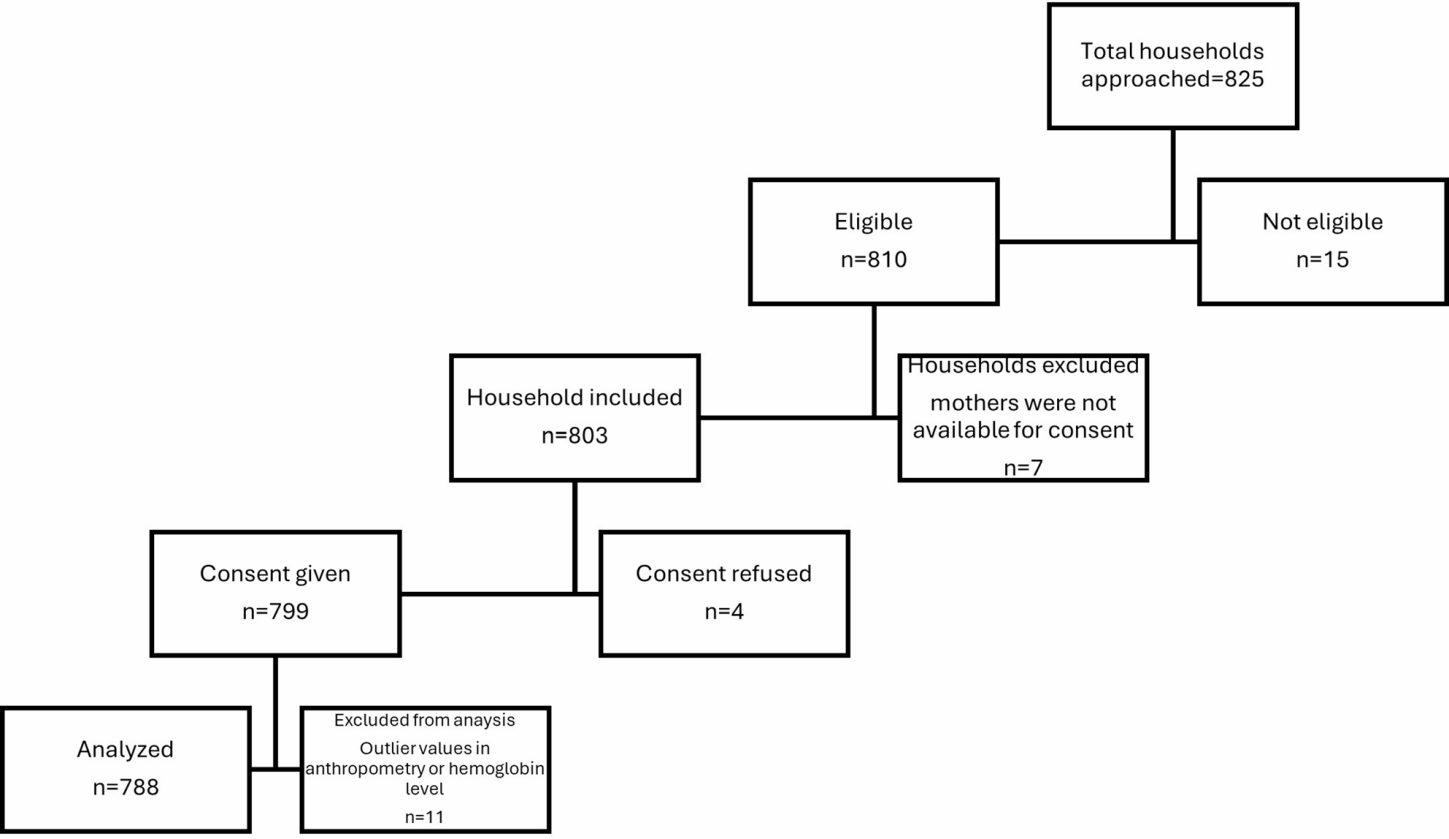
Participants flow chart

Overall, the research team approached 825 households to recruit eligible participants. Out of 825 households, 810 households had 10-19-years of age individual that fulfilled the eligibility criteria. However, 7 households were excluded due to unavailability of the mother for consent. In 803, households, assent and consent were successfully obtained from 799 participants. The data from 799 rural households and adolescents were collected and data of eleven participants excluded due to implausible anthropometric values. Data of 788 adolescents were analyzed for study outcomes (Figure: 1).

Table: 1 shows the distribution of sociodemographic factors in adolescents overall, and among stunted, thin, and anaemic individuals. There were 399 (50.4%) boys out of 788 participants. The mean age was 13.5 years with a standard deviation of 2.7 years. About a quarter (*n* = 212 (26.9%) never attended school and nearly half (381 [48.4%]) have < 3 years of schooling, and half of the adolescents were food insecure. Nearly half (45.8%) of the fathers and 85% of the mothers were uneducated.

**Fig. 2:**
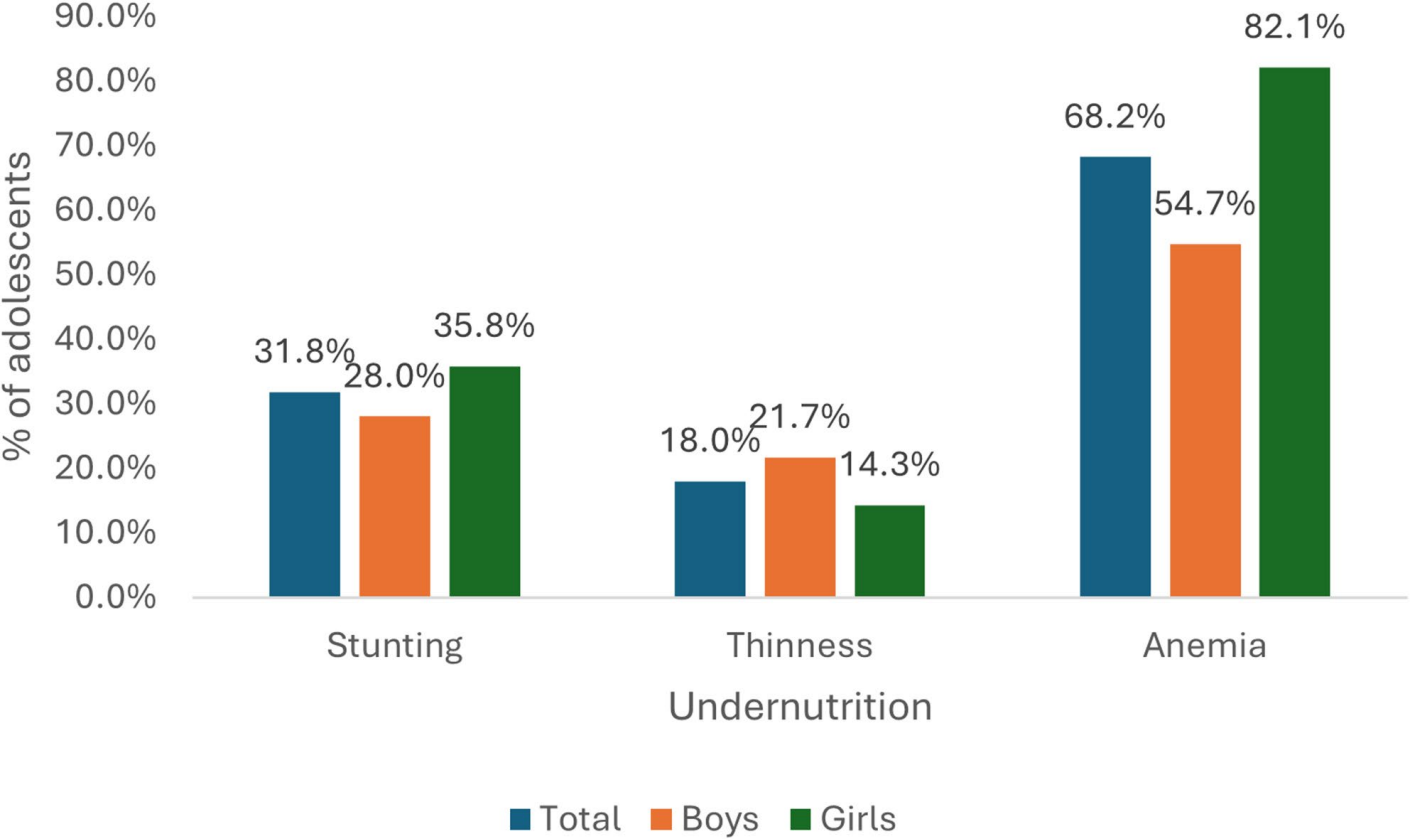
Distribution of stunting, thinness, and anemia among adolescent boys and girls

Figure: 2 shows that about two-thirds of the sample (68.2%) were anemic, 32% were stunted and 18% were thin. Stunting and anemia were higher among girls (35.8% vs. 28.0%; 82.1% vs. 54.7% respectively) compared to boys. More boys were thin (21.7 vs. 14.3%) compared to girls.

The daily intake of different food groups by adolescent girls and boys is displayed in Table 2. In our sample, grains, roots, and tubers (GRT) were the most consumed group (99.7%). Within this food group, *roti* (flat bread) was consumed by almost all (96.3%) of the participants, rice was taken only by 15% and among tubers and plantains, the potato was the commonest tuber taken by 11.3%.

**Table 1:** Distribution of socio-demographic factors among adolescents with stunting, thinness and anemia

Variables	Total *n* = 788 *n* (%) Mean ± SD	Stunting *N* = 251(31.8%) *n* (%) Mean ± SD	Thinness *n* = 142 (18.0%) *n* (%) Mean ± SD	Anemia *n* = 538 (68.2%) *n* (%) Mean ± SD
Boys	397 (50.4)	111 (28.0)	86 (21.7)	217 (54.7)
Girls	391 (49.6)	140 (35.8)	56 (14.3)	321 (82.1)
Food secure adolescents	373 (47.3)	117 (46.6)	56 (39.4)	269 (50.0)
Food insecure adolescents	415 (52.7)	134 (53.4)	86 (60.6)	269 (50.0)
Food secure households	479 (60.8)	150 (59.8)	78 (54.9)	323 (60.0)
Food insecure households	309 (39.2)	101 (40.2)	64 (45.1)	215 (40.0)
Adolescents’ age groups				
Early (10–14 years)	485 (61.5)	143 (29.5)	89 (18.4)	346 (71.3)
Late (15–19 years)	303 (38.5)	108 (43.0)	53 (37.3)	192 (35.7)
Adolescents’ education				
No schooling	212 (26.9)	74 (34.9)	31 (14.6)	168 (79.2)
1–5 years	195 (24.7)	60 (30.8)	40 (20.5)	140 (71.8)
> 5 years	381 (48.4)	117 (30.7)	71 (18.6)	230 (60.4)
Father’s education				
Illiterate	361 (45.8)	119 (47.4)	64 (45.1)	264 (49.1)
Literate	427 (54.2)	132 (52.6)	78 (54.9)	274 (50.9)
Mother’s education				
Illiterate	667 (84.6)	215 (85.7)	123 (86.6)	458 (85.1)
Literate	121 (15.4)	36 (14.3)	19 (13.4)	80 (66.1)
Number of male siblings	2.9 ± 1.5	2.8 ± 1.6	2.8 ± 1.5	2.9 ± 1.6
Number of female siblings	2.4 ± 1.5	2.2 ± 1.5	2.4 ± 1.6	2.4 ± 1.5
Total number of siblings	2.2 ± 1.5	2.0 ± 1.5	2.1 ± 1.6	2.1 ± 1.5
Wealth quintiles				
Poorest	194 (24.6)	77 (30.7)	41 (28.9)	137 (25.5)
Poor	209 (26.5)	68 (27.1)	42 (29.6)	149 (27.7)
Middle	78 (9.9)	21 (8.4)	14 (9.9)	51 (9.5)
Rich	152 (19.3)	41 (16.3)	22 (15.5)	98 (18.2)
Richest	155 (19.7)	44 (17.5)	23 (16.2)	103 (19.1)

**Table 2:** Daily dietary intake of the adolescents living in the rural area of Sindh

Food groups	Total *N* = 765 *n* (%)	Boys *N* = 388 *n* (%)	Girls *N* = 377 *n* (%)	*p*-value
Grains, white roots and tubers, and plantains	763 (99.7)	388 (100)	375 (99.5)	0.15
Pulses (beans, peas, and lentils)	13 (1.7)	6 (1.5)	7 (1.9)	0.74
Nuts and seeds	0 (0.0)	0 (0.0)	0 (0.0)	-
Dairy	454 (59.3)	256 (66.0)	198 (52.5)	< 0.01
Meat, poultry, and fish	0 (0.0)	0 (0.0)	0 (0.0)	-
Eggs	5 (0.7)	3 (0.8)	2 (0.5)	1.00
Dark green leafy vegetable ^#^	5 (0.7)	4 (1)	1 (0.3)	0.37
Other vitamin A-rich fruits and vegetables	159 (20.8)	91 (23.5)	68 (18.0)	0.06
Other vegetables	144 (19.5)	115 (31.3)	29 (7.8)	< 0.01
Other fruits	53 (6.9)	15 (3.9)	38 (10.1)	< 0.01
Fried snacks	148 (19.3)	61 (15.7)	87 (23.1)	< 0.01
Sugar-sweetened beverages	742 (97.0)	383 (98.7)	359 (95.2)	< 0.01
Sweets	453 (59.2)	248 (63.9)	205 (54.4)	< 0.01

*n = 765; #Fisher Exact test was used

**Table 3:** Multivariable model for factors associated with stunting, thinness, and anemia among adolescent girls and boys residing in rural Sindh

Variables *N* = 788	Stunting		Thinness		Anemia	
	Crude Prevalence ratio (95% CI)	Adjusted Prevalence ratio (95%CI)	Crude Prevalence ratio (95% CI)	Adjusted Prevalence ratio (95%CI)	Crude Prevalence ratio (95% CI)	Adjusted Prevalence ratio (95%CI)
Boys	1	1	1	1	1	1
Girls	1.06(0.94,1.19)	1.34(1.03,1.74)	0.92(0.88,0.98)	0.72(0.48,1.10)	1.31(1.23,1.40)	1.34(1.12,1.61)
Food secure adolescents	1	-	1	1	1	-
Food insecure adolescents	1.02(0.80,1.31)		1.38 (0.98,1.93)	1.07(0.67,1.69)	0.93(0.79,1.10)	
Food secure households	1	-	1	1	1	-
Food insecure households	1.04(0.81,1.34)		1.27(0.91,1.77)	1.11(0.73,1.68)	1.09(0.92,1.29)	
Adolescents’ age groups						
Early (10–14 years)	1	1	1	1	1	1
Late (15–19 years)	1.20 (0.94, 1.55)	1.26(0.97, 1.64)	0.95 (0.67,1.33)	1.15(0.77, 1.71)	0.88(0.67, 1.15)	0.97 (0.71, 1.32)
Father’s education						
No schooling	1	-	1	-	1	1
At least one year of schooling	0.93 (0.73, 1.20)		1.03(0.74,1.43)		1.32(1.01,1.71)	1.18(0.88,1.57)
Mother’s education						
Illiterate	1	1	1	1	1	1
Literate	1.01(0.9,1.0)	0.90(0.62,1.31)	1.02(0.61,1.69)	0.98(0.59,1.64)	1.2(0.7, 1.6)	1.20(0.83,1.72)
Adolescents’ education						
No schooling	1	-	1	1	1	1
1–5 years	0.88(0.65,1.18)		1.39(0.92,1.12)	1.43(0.89,2.29)	0.82(0.68,1.00)	1.25(0.84,1.85)
> 5 years	0.87(0.62,1.22)		1.17(0.72,1.90)	1.31(0.76,2.27)	0.79(0.63,0.99)	1.34(0.87,2.06)
Number of male siblings	0.96(0.88,1.04)	-	0.97(0.87,1.08)	-	1.00(0.95,1.06)	-
Number of female siblings	0.93(0.85,1.00)	0.96(0.85,1.08)	0.99(0.89,1.00)	-	1.01(0.96,1.07)	-
Total number of siblings	1.02(0.93,1.12)	0.95(0.88,1.04)	1.02(0.95,1.10)	0.97(0.90,1.05)	1.01(0.87,1.18)	0.97(0.91,1.03)
Wealth quintiles						
Poorest	1.39(0.96, 2.02)	1.42(0.96,2.09)	1.42(0.85, 2.37)	1.46(0.82,2.60)	0.87(0.60, 1.26)	1.00(0.66,1.52)
Poor	1.14(0.78, 1.67)	1.15(0.77,1.70)	1.35(0.81, 2.25)	1.39(0.80,2.41)	0.74(0.50, 1.08)	0.83(0.56,1.25)
Middle	0.88 (0.56, 1.36)	0.87(0.55,1.37)	1.31 (0.75, 2.27)	1.28(0.71,2.29)	0.89 (0.59, 1.33)	0.91(0.59,1.38)
Rich	1.06 (0.65, 1.72)	1.01(0.61,1.65)	0.62 (0.28, 1.40)	0.55(0.23,1.30)	0.95 (0.60, 1.50)	0.98(0.61,1.52)
Richest	1	1	1	1	1	1

Models were adjusted for the adolescent and household-level FI, age of adolescents, education of mother/father, number of siblings and wealth quintiles.

The daily consumption of vegetables and fruits was overall low, and vegetable intake was higher compared to fruits. Among vegetables, the least consumed vegetable was dark green leafy vegetables (0.7%). More than half (59.3%) of the adolescents’ daily diet included dairy and dairy products. The intake of pulses was only 1.7%, none of the participants were taking any kind of meat and nuts daily and only 0.7% consumed eggs daily.

We found a higher intake of unhealthy foods such as sugar-sweetened beverages (97.0%) and sweets and confectionaries (59.2%). Sugar-sweetened tea was the most common beverage taken by 92.5% of the participants. About 19.0% of adolescents were taking fried snacks. More boys were found to be consuming dairy and vegetables than girls (p-value < 0.05). Girls were found to indulge more in fried snacks and fruits (p-value < 0.01).

**Fig. 3:**
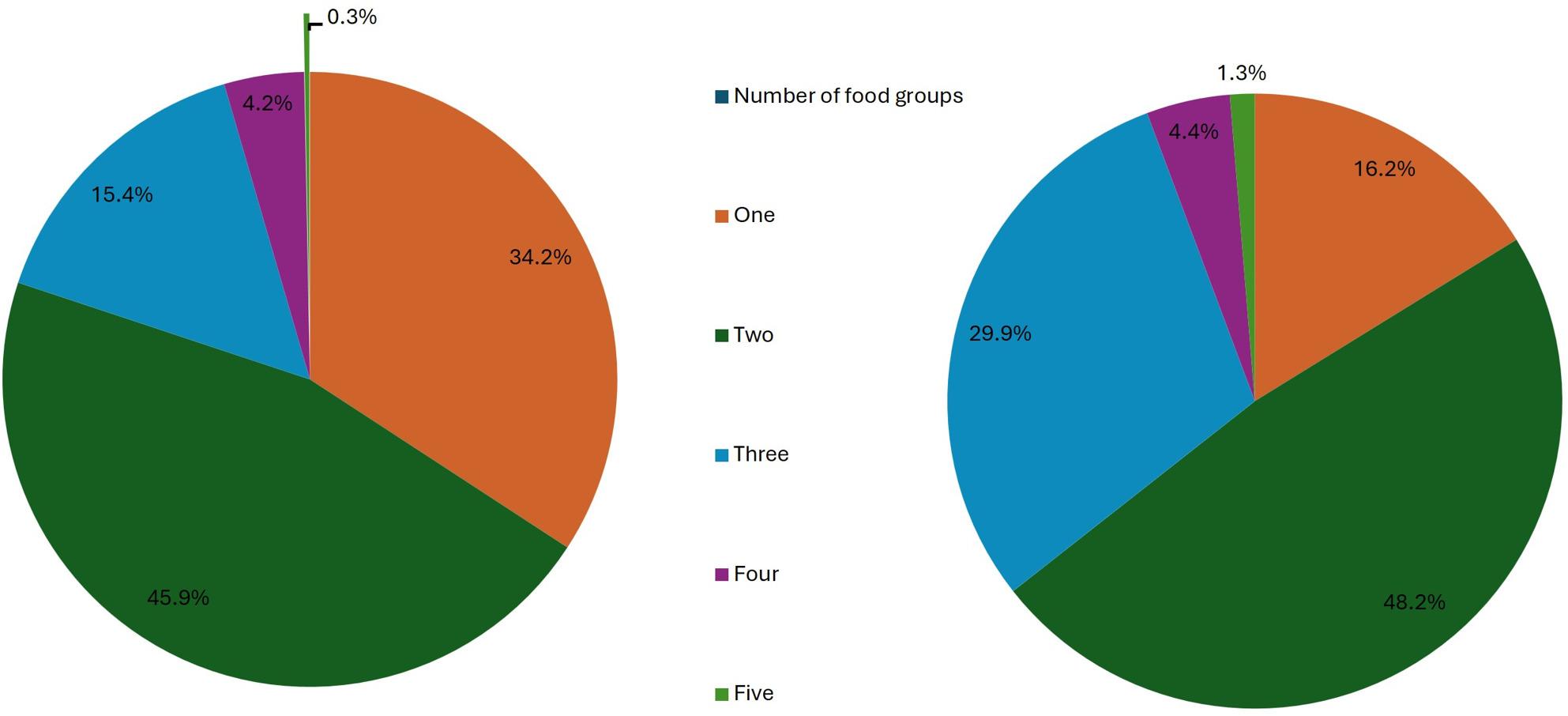
Number of food groups intake among adolescent girls and boys

Figure 3 depicts the proportion of adolescents consuming the number of food groups in their daily diet. The MDD, i.e. consumption of at least 5 food groups out of 10 groups recommended by FAO was found only in 0.8% of adolescents. When MDD was compared between rural boys and girls, no difference was found (boys *n* = 5 (1.2%), girls *n* = 1 (0.2%); p-value 0.21). 94% of boys and 95.4% of girls consumed only 1–3 food groups daily.

Table: 3 demonstrates the multivariable models after adjusting for stunting, thinness, and anemia. Gender was the only significant variable in the adjusted model for stunting. Stunting was more prevalent among girls compared to boys (APR 1.34 95% CI [1.03, 1.74]). The prevalence of thinness among girls was 28% less compared to boys at the multivariable level however it was statistically insignificant (95% CI 0.48, 1.10). None of the variables were significantly associated with thinness in our data. Similar to stunting, only gender was associated with anemia. Girls were 1.34 times more likely to be anemic than boys (95% CI 1.12, 1.61) after adjusting for the age of adolescents, education of mother/father, number of siblings and wealth quintiles.

As gender was significantly associated with stunting and anemia, therefore gender stratified analysis was undertaken as instructed by Canadian Institutes of Health Research (CIHR) to investigate the variables that might be differently associated with undernutrition among boys and girls [38]. The gender-stratified valid estimates are presented in Table 4 (for girls) and Table 5 (for boys) for stunting, thinness, and anemia.

**Table 4:** Multivariable model for stunting, thinness, and anemia in adolescent rural *girls*

Variables *N* = 391	Stunting		Thinness		Anemia	
	Crude Prevalence ratio (95% CI)	Adjusted Prevalence ratio (95%CI)	Crude Prevalence ratio (95% CI)	Adjusted Prevalence ratio (95%CI)	Crude Prevalence ratio (95% CI)	Adjusted Prevalence ratio (95%CI)
Food secure adolescents	1	-	1	-	1	**-**
Food insecure adolescents	1.18 (0.82, 1.69)		1.30 (0.74,2.29)		1.03 (0.80, 1.32)	
Food secure household	1	-	1	-	1	-
Food insecure household	1.16(0.81,1.65)		1.31(0.76,2.27)		1.08(0.85,1.37)	
Adolescents’ age groups						
Early (10–14 years)	1	1	1	-	1	-
Late (15–19 years)	1.28(0.91,1.78)	1.42(1.01,2.00)	0.87(0.51,1.49)		1.08(0.86,1.35)	
Father’s education						
Illiterate	1	-	1	-	1	-
Literate	0.83(0.60,1.16)		1.14(0.67,1.95)		0.97(0.78,1.21)	
Mother’s education						
Illiterate	1	-	1	-	1	-
Literate	0.84(0.54,1.30)		1.17(0.63,2.18)		0.91(0.69,1.21)	
Adolescents’ education						
No schooling	1	-	1	-	1	-
1–5 years	0.81(0.55,1.18)		1.29(0.70,2.37)		0.89(0.69,1.14)	
> 5 years	0.80(0.52, 0.25)		1.29(0.64,2.57)		0.90(0.67,1.20)	
Number of male siblings	0.97 (0.87, 1.08)	-	0.97 (0.82, 1.15)	-	1.02 (0.95, 1.09)	-
Number of female siblings	0.91(0.81,1.01)	0.91(0.82,1.02)	0.97(0.82,1.15)	-	1.01(0.94,1.08)	-
Wealth quintiles						
Poorest	1.71(1.05,2.77)	1.81(1.11,2.97)	1.78(0.82,3.85)		1.01(0.73,1.41)	
Poor	1.08(0.65,1.81)	1.12(0.67,1.88)	1.45(0.66,3.17)		1.06(0.77, 0.45)	
Middle	1.15(0.65,2.06)	1.14(0.67,1.88)	1.14(0.45,2.91)		1.00(0.69,1.44)	
Rich	1.11(0.59,2.10)	1.08(0.57,2.05)	0.58(0.16,2.10)		1.02(0.68,1.52)	
Richest	1	1	1	-	1	-

Models were adjusted for the adolescent and household-level FI, age of adolescents, education of mother/father, number of siblings and wealth quintiles.

Adjusted model in Table: 4 showed that among girls, stunting was more prevalent in the 15–19 years of age group compared to the younger adolescent (APR 1.42 95% CI 1.01, 2.00). Stunting was 1.81 times more prevalent in the girls belonging to the poorest households compared to the girls from the richest households. Thinness and anemia were not significantly associated with any of the studied factors in stratum-specific multivariable analysis.

Unlike girls, stratum-specific multivariable analysis in Table: 5 depicted that among boys, none of the studied factors were associated with stunting, thinness, and anemia.

**Table 5:** Multivariable model for stunting, thinness, and anemia in adolescent rural *boys*

Variables *N* = 397	Stunting		Thinness		Anemia	
	Crude Prevalence ratio (95% CI)	Adjusted Prevalence ratio (95%CI)	Crude Prevalence ratio (95% CI)	Adjusted Prevalence ratio (95%CI)	Crude Prevalence ratio (95% CI)	Adjusted Prevalence ratio (95%CI)
Food secure adolescents	1	-	1	-	1	1
Food insecure adolescents	1.33 (0.81, 2.19)		1.04 (0.62, 1.75)		1.32 (0.94, 1.85)	1.24 (0.86, 1.77)
Food secure household	1	-	1	-	1	1
Food insecure household	1.03(0.71,1.50)		1.10(0.72,1.69)		1.26(0.97,1.63)	1.14(0.86,1.51)
Adolescents’ age groups						
Early (10–14 years)	1	-	1	-	1	-
Late (15–19 years)	1.28(0.91,1.78)		1.07(0.68,1.66)		0.94(0.72,1.24)	
Father’s education						
Illiterate	1	-	1	-	1	1
Literate	1.08(0.74,1.58)		0.95(0.62,1.45)		0.79(0.61,1.02)	0.81(0.62,1.06)
Mother’s education						
Illiterate	1	-	1	-	1	-
Literate	0.95(0.51,1.78)		0.65(0.28,1.49)		0.75(0.47,1.20)	
Adolescents’ education						
No schooling	1	-	1	-	1	-
1–5 years	1.49(0.79,2.83)		1.09(0.58,2.05)		0.89(0.62,1.28)	
> 5 years	1.48(0.75,2.92)		0.85(0.42,1.72)		0.79(0.53,1.19)	
Number of male siblings	0.97(0.87,1.08)	-	0.94(0.82,1.08)	-	1.01(0.93,1.10)	-
Number of female siblings	0.95(0.84,1.07)	-	1.01(0.88,1.15)	-	1.02(0.94,1.10)	-
Wealth quintiles						
Poorest	1.12(0.63, 0.01)		1.12(0.56, 0.21)		1.26(0.83,1.94)	1.04(0.66,1.65)
Poor	1.22(0.69, 0.15)		1.20(0.61,2.36)		1.35(0.89,2.06)	1.19(0.77,1.84)
Middle	0.67(0.33, 0.33)		1.23(0.61,2.48)		1.28(0.82,1.99)	1.13(0.72,1.79)
Rich	1.00(0.47,2.13)		0.63(0.22,1.78)		1.08(0.62,1.87)	1.02(0.58,1.77)
Richest	1	-	1	-	1	1

Models were adjusted for the adolescent and household-level FI, age of adolescents, education of mother/father, number of siblings and wealth quintiles.

## Discussion

The nutritional status of adolescent girls and boys was alarming. The daily diet of girls and boys did not include essential foods like eggs and meat and < 1% achieved MDD. We are one of the first ones to report community-based rate of anemia of adolescent rural boys in Pakistan. Anemia was highly prevalent in girls and boys, and it was significantly higher in girls. About a third of the sample was stunted and more girls were stunted than boys. Thinness was higher in boys than girls. Gender was significantly associated with anemia and stunting. No other socio-demographic variables were associated with undernutrition and anemia.

The estimates of anemia in girls (82.1%) in our study were much higher than the rural Sindh estimates (63.1%) reported by NNS 2018 whereas the burden of stunting and thinness in girls and boys in our study was similar to the prevalence documented by NNS 2018 [14]. Most of the anemia, stunting and thinness estimates in adolescent boys in Pakistan are from schools based, urban area studies [39–42]. The limited literature on rural boys reported high rates of undernutrition. Campisi et al. reported 27% stunting, and 31% thinness among boys in rural Sindh which was coherent with our findings [43].

As for anemia, Baxter et al. reported lower rates than our study rates of anemia. The Matiari emPowerment and Preconception Supplementation (MaPPS) trial documented 53.6% anemia in late adolescent girls. It should be noted that 13.3% of the MaPPS cohort achieved MDD whereas a negligible percentage of MDD was found in our sample [44]. Our high rates of anemia correlated with < 1% of girls consuming any kind of meat, eggs, and dark green leafy vegetables daily. The high rate of anemia is plausible with the given diet pattern because such diet can lead to deficiency of iron and vitamins which are essential for preventing anemia [45].

We found higher odds of anemia and stunting in girls than boys. Due to the lack of Pakistani literature on gender differences in anemia and stunting, we cannot confirm or contrast the gender difference found in our study. However, supporting our results, a trend analysis on iron deficiency anemia among south Asian adolescents and disability-adjusted life years (DALYs) showed 1400 DALYs were from 15-19-year-old girls, and 600 DALYS from boys of the same age group per 100,000 adolescents [46]. Similar higher odds of anemia in girls were reported from rural China compared to boys (OR 1.73 (95% CI 1.21, 2.48) [47]. These findings support the gender difference in anemia found in our study.

We found significant gender differences in stunting with more girls being stunted than boys. Our results were contradictory to the pooled analysis from African countries and Brazilian study where more boys were stunted [48, 49]. However, consistent with our results, higher rates of stunting among girls were reported in Bangladesh (43.1% boys vs. 50.3% girls ) and other studies from Pakistan (27.0% boys vs. 37.2% girls) [26, 43]. Biologically linear growth in girls ceases after puberty which is attained earlier than boys [50, 51]. The biological explanation of higher stunting in our sample can be the early puberty in south Asians [12, 26]. Moreover, chronic inadequate diet can cause stunting. Gender discrimination in food distribution is well-documented in patriarchal societies like Pakistan where boys are preferred over girls [21, 52].

Similar to our results, NNS 2018 reported 20% boys to be underweight compared to 11% of girls [14]. The pattern of girls weighing more than boys at puberty had been observed in African studies [9, 12, 53]. Physiologically girls reach sexual maturation earlier than boys and store more fat mass compared to boys who store more lean mass [12]. Also compared to boys, the physical activity of adolescent girls gets restricted due to cultural and social norms in LMICs like Pakistan, especially in rural areas and that contributes to weight gain in girls [53].

Other than gender we did not find any socio-demographic factors associated with undernutrition and anemia. Aligned with our findings, the MaPPS trial did not find any of the social determinants of health (such as wealth status, household FI, parents’ education, and occupation) to be associated with anemia in girls [44]. A systematic review on the association of household-level FI and anemia in adolescents, did not find significant results (OR = 1·08; 95% CI 0·71, 1·44) [54]. Nonetheless, contrary to our findings, a rural Indian study reported, an increase in anemia with poor diet diversity [55]. MDD proportion was too low in our sample to determine the statistical association of it with anemia. Moreover, anemia was associated with age of the adolescent [56] in India, and the educational status of mothers [47], and wealth status [47] in young school-going adolescents in rural China and Ethiopia.

Similar to our results, Irenso et al. did not report association of socio-demographic factors with stunting in Ethiopia [57] and in Bangladesh, early adolescence was protective against thinness in girls and no other factor was significant [58]. A couple of studies found increase in stunting and thinness with lower wealth status [59], and household size [60], and demonstrated the negative effect of FI on the gain in height during adolescence [61].

Overall, our findings were not aligned with the literature. It could be due to the difference in our study population. We included both boys and girls, 10–19 years of age, both school-attending and non-attending, whereas the mentioned studies mainly had early-age and school-going adolescents. These differences in the study population in our sample and literature may have altered the association of reported factors of undernutrition and anemia in our study. Another reason can be the use of prevalence ratio to find association between variables instead of odds ratio which is commonly reported by the mentioned studies. Due to the inflation of the estimates, the odds ratio incorrectly finds a significant association when there is none [36]. PR provides more robust estimates hence our results are different from other studies as others reported ORs whereas we have used a more robust statistical measure and reported PRs.

### Strengths and limitations

The main highlight of the study is that it is a community-based study that recruited both school-going and out-of-school girls and boys across the whole age span of adolescence. We consider community-based data our strength because half of the Pakistani adolescents are out-of-school and school-based studies are likely to undermine the burden of the problem as out-of-school adolescents are likely to be from the poorest wealth quintile and more vulnerable to undernutrition and anemia [62].

Another strength was a better measure of association in cross-sectional design than odds ratio. Odds ratios provide overinflated estimates when use for prevalent outcomes and may find associations in the cross-sectional study when the variables are not associated.

This work has some limitations as well that needed to be considered while interpreting the results. The selection of rural communities was based on logistic convenience which could have introduced selection bias. However, we anticipate that any under or overestimation would have been random i.e. equally divided in the sample.

One limitation of the study was the use of FFQ instead of 24-hour dietary recalls at multiple time points. It is preferred to use 24-hour recall to measure dietary diversity covering the seasonal variations and differences in diet during weekdays and weekends by collecting dietary data multiple times. The study design, i.e. cross-sectional survey did not allow collecting serial data. However, the reference range of FFQ recall is 12 months but considering the limitation we anticipate that dietary diversity would have been underestimated for all the participants because seasonal variability was not captured due to the nature of the data collection tool.

Another shortcoming of our work is the inability to identify the cause-and-effect relationship between the studied factors and undernutrition and anemia. The cross-sectional study design limits inference to associations only, not causation, as explicitly reflected in the PRs reported. The ideal design would have been a prospective cohort study starting at an early age and following the participants into their adolescence. However, the cost and time constraints did not allow for conducting a prospective study. We adjusted for commonly reported confounders of undernutrition and anemia to avoid spurious associations but there could have been residual confounding due to variables that we have not considered in our analysis. Future studies should also explore the nutrition and digital literacy of young group to design nutrition responsive interventions.

## Conclusion

The high prevalence of stunting shows chronic undernutrition and missing the second window of opportunity during adolescence to gain potential adult height. Optimum nutrition and health care are important to get benefits from the growth spurt in adolescence. Still, we observed a very high rate of low dietary diversity among adolescents, which was reflected in the alarmingly high anemia rates in the sampled adolescents. The lack of nutrition diversity and poor nutritional status in this critical phase of life can limit the lifelong potential of physical, mental, economic, and social well-being of millions of girls and boys. The short-term success to control undernutrition and anemia can be providing food supplements, free meals, and multivitamins to both girls and boys. For sustainable improvements, food fortification programs and poverty alleviation interventions should be strengthened.

## Data Availability

The datasets used and/or analysed during the current study are available from the corresponding author on reasonable request.
